# Optimizing the single-step model for predicting fumonisins resistance in maize hybrids accounting for the genotype-by-environment interaction

**DOI:** 10.3389/fgene.2025.1475452

**Published:** 2025-07-02

**Authors:** Jeniffer Santana Pinto Coelho Evangelista, Kaio Olimpo das Graças Dias, Maria Marta Pastina, Saulo Chaves, Lauro José Moreira Guimarães, Jorge Hidalgo, Julian Garcia-Abadillo, Reyna Persa, Valéria Aparecida Vieira Queiroz, Dagma Dionísia da Silva, Leonardo Lopes Bhering, Diego Jarquin

**Affiliations:** ^1^ Agronomy Department, University of Florida, Gainesville, FL, United States; ^2^ Departamento de Biologia Geral, Universidade Federal de Viçosa, Campus Universitário, Viçosa, Minas Gerais, Brazil; ^3^ Embrapa Milho e Sorgo, Sete Lagoas, Minas Gerais, Brazil; ^4^ Departamento de Genética, Escolha Superior de Agricultura “Luiz de Queiroz”, Universidade de São Paulo, São Paulo, Brazil; ^5^ Department of Animal and Dairy Science, University of Georgia, Athens, GA, United States; ^6^ Centro de Biotecnología y Genómica de Plantas, Universidad Politécnica de Madrid (UPM), Madrid, Spain

**Keywords:** fumonisins resistance, genomic prediction, plant breeding, maize hybrid prediction, single-step model

## Abstract

In Brazil, disease outbreaks in plant cultivars are common in tropical zones. For example, the fungus *Fusarium verticillioides* produces mycotoxins called fumonisins (FUMO) which are harmful to human and animal health. Besides the genetic component, the expression of this polygenic trait is regulated by interactions between genes and environmental factors (G × E). Genomic selection (GS) emerges as a promising approach to address the influence of multiple loci on resistance. We examined different manners to conduct the prediction of FUMO contamination using genomic and pedigree data, and combinations of these two via the single step model (**B**-matrix) which also offers the possibility of increasing training set sizes. This is the first study to apply the **B**-matrix approach for predicting FUMO in tropical maize breeding programs. Our research introduced a cross-validation approach to optimize the hyper-parameter *w*, which represents the fraction of total additive variance captured by the markers. We demonstrated the importance of selecting optimal w by environment in unbalanced datasets. A total of 13 predictive models considering General Combining Ability (GCA) and Specific Combining Ability (SCA) effects, resulted from five linear predictors and three different covariance structures including the single-step approach. Two cross-validation scenarios were considered to evaluate the model’s proficiency: CV1 simulated the prediction of completely untested hybrids, where the individuals in the validation set had no phenotypic records in the training set; and CV2 simulated the prediction of partially tested hybrids, where individuals had been evaluated in some environments but not in the target environment. Results showed that using the **B**-matrix in the five tested linear models increased the predictive ability compared to pedigree or genomic information. Under CV1, increasing training set sizes exhibit superior predictive accuracy. On the other hand, under CV2 the advantages of increasing the training set size are unclear and the improvements are due to better covariance structures. These insights can be applied to plant breeding programs where the GCA, SCA, and G × E interactions are of interest and pedigree information is accessible, but constraints related to genotyping costs for the entire population exist.

## 1 Introduction

In tropical environments such as in Brazil, outbreaks of pests and diseases are more frequent and can exhibit significant variations between locations, years, and seasons within the same year, directly affecting crop productivity (Oliveira et al., 2014; Chaves et al., 2023). Among the most frequent diseases in corn crops, ear rot, caused by the fungus *Fusarium verticillioides*, is one of the most prevalent and economically significant diseases (Jorge et al., 2022). In addition to causing losses in productivity, the fungus produces mycotoxins. Fumonisins (FUMO) are particularly concerned due to their widespread occurrence and significant human and animal health impacts (Blacutt et al., 2018). FUMO are a class of mycotoxins that mainly affect maize grains and can contaminate derived products, such as maize flour (Butoto et al., 2022). Therefore, it is of utmost importance that maize breeding programs consider plant resistance to FUMO contamination as a selection criterion (Lanubile et al., 2014; Holland et al., 2020).

However, phenotyping this trait can be expensive and time-consuming (Bush et al., 2004). Hence, the genomic prediction (GP) emerges as an important alternative strategy for evaluating maize cultivars for resistance to fumonisin contamination. Potentially, this approach could help to increase genetic gains by reducing the required time and associated costs in identifying the most promising materials (Heslot et al., 2015). The GP involves developing predictive models by integrating phenotypic and genomic information derived from single nucleotide polymorphisms (SNPs) - molecular markers. These models are then applied to estimate the genetic potential of individuals whose phenotypes have not been measured, based only on their marker profiles (Meuwissen et al., 2001). Genomic selection (GS), the breeding process that applies GP in breeding decisions, can be particularly useful in hybrid breeding. In GS models for hybrids, the effects of General Combining Ability (GCA) and Specific Combining Ability (SCA) have been widely used (Acosta-Pech et al., 2017; Jarquin et al., 2021; Fonseca et al., 2021; Zhang et al., 2022; Melchinger and Frisch, 2023). The GCA refers to the average performance of a parent in producing desirable traits in its progeny when crossed with different parents, while SCA reflects the specific interaction between two parents, indicating their compatibility and ability to produce superior progeny.

GS offers the opportunity to increase the selection intensity, expedite the breeding cycles, increase the genetic gains, and enable the efficient allocation of resources in breeding programs (Atanda et al., 2021; Beyene et al., 2021; Persa et al., 2021). These advantages have led many breeding companies to incorporate GS into their programs, including efforts focused on fungal resistance traits. Recent research has demonstrated the potential of GS for improving resistance to fumonisin contamination in maize, highlighting its efficiency and cost-effectiveness compared to traditional phenotypic selection (Butoto et al., 2022). GS exploits the realized genomic relationships between genotypes based on the proportions of alleles they share through the genetic matrix (**G**; VanRaden et al., 2008), whose entries describe the genomic similarities between pairs of individuals. This method provides a more accurate representation of genetic inheritance by accounting for Mendelian sampling, enabling the detection of genetic differences between individuals with identical expected similarities. In contrast, pedigree-based selection relies on a relationship matrix (**A**), constructed solely on the expected similarity between individuals (Hayes et al., 2009).

This does not mean that the pedigree information is expendable or lacks value in data analysis. Frequently, not all the selection candidates or parental lines are genotyped, but their pedigree is registered (Callister et al., 2021). In this scenario, **G** (the matrix of genomic relationships) can be enriched or complemented by **A** (the pedigree matrix) forming a single relationship matrix $B=w\times A+\left(1‐w\right)\times G$ resulting in the well-known single step model (Misztal et al., 2009; Aguilar et al., 2010). In other words, the **B**-matrix combines genomic and pedigree information with a weighting factor/hyper-parameter (*w*). The *w* hyper-parameter represents the fraction of the total additive variance not captured by the markers (Velazco et al., 2019). To find the best way to build the **B**-matrix, different values for *w* are considered, and the value that returns the highest predictive ability in training sets is chosen for performing the prediction of individuals in the testing set (Velazco et al., 2019; Oliveira et al., 2020).

In addition, the single step approach allows combining the full set of genotypes (genotyped and non-genotyped but with pedigree information only) using a standard genomic selection method resulting in an increased training set size. The Genomic Best Linear Unbiased Predictor (GBLUP) linear predictor can be used to implement the single-step GBLUP, or ssGBLUP (Legarra et al., 2014) approach. Several studies highlight the potential of ssGBLUP compared to the traditional GBLUP and ABLUP (pedigree-based selection) for predicting untested genotypes (Ashraf et al., 2016; Ukrainetz and Mansfield, 2020).

In this study, we analyzed a dataset consisting of 373 single-cross tropical maize hybrids derived from 359 inbred lines. These hybrids were evaluated over 3 years, with each year’s assessment taking place in the same location resulting in three different environments (year-by-location combination). The objective was to evaluate the effectiveness of the single-step approach (referred to as the **B**-matrix) in maize breeding programs aimed at reducing FUMO levels in grains. Wherein the deployment of GP considered the following aims: *i*) optimization of the election of the *w* hyper-parameter to combine genomic information and pedigree data in prediction models via the **B**-matrix, where *w* represents the proportion of total additive genetic variance explained by markers; *ii*) comparing the predictive ability of different models based on main and interaction effects; SNPs matrix (**G**), pedigree information (**A**), and the hybrid matrix (**B**) via the single-step model, *iii*) evaluating the impacts on predictive ability by increasing training set sizes using phenotyped individuals with pedigree information only.

## 2 Materials and methods

### 2.1 Phenotypic data

**FIGURE 1:**
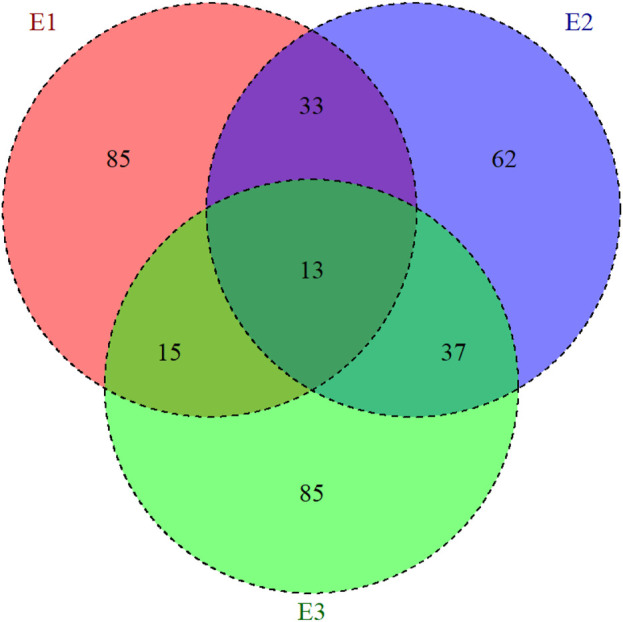
Representation of the allocation of the maize hybrids evaluated in three environments (E1, E2, and E3). The inner circle presents the common number of hybrids that were evaluated by pairs of environments.

The analyzed datasets correspond to three maize trials established at the Brazilian Agricultural Research Corporation (EMBRAPA) Maize and Sorghum headquarters in Sete Lagoas city at the Minas Gerais state, Brazil (19°28′S, 44°15′W). These trials were conducted in three consecutive agronomical years (2014/2015, 2015/2016, and 2016/2017), and each agronomical year was considered as a different environment (E1 = 2014/2015 year; E2 = 2015/2016 year, and E3 = 2016/2017 year). Genotypes were planted in a lattice design, with two repetitions. In total 373 single-cross hybrids were evaluated: 146 in environment 1 (E1), 145 in E2, and 150 in E3. Furthermore, care was taken to allow connectivity between environments: 33 hybrids are common between E1 and E2, 15 between E1 and E3, 37 between E2 and E3, and 13 across the three environments (Figure 1).

All 373 single-cross hybrids were scored for fumonisin concentration in parts per million (ppm) (FUMO), and this information was missing for the parents of these hybrids. The determination of the FUMO trait was initially based on a 500 g sample of corn grains that required a meticulous quantification process. The quantification took place at the Laboratory of Food Safety at Embrapa Milho e Sorgo where a 10 g subsample was finely ground. Then fumonisins were extracted in a solution of 100 mL water/methanol mixture (20/80), and 5 g NaCl using a blender for 1 min. The resulting mixture was then filtered through Whatman paper, and an aliquot of 10 mL of the filtered extract was subsequently diluted in 40 mL of 0.1% phosphate Tween-20 solution (phosphate buffer). This solution was then filtered again using a 1.0 mm microfiber filter. Then a subsample of 10 mL of this solution was passed through the FumoniTest column, which was washed with 10 mL of phosphate buffer solution followed by a second flow of 10 mL of phosphate buffer. The contents of the column were eluted with 1.0 mL of methanol (HPLC grade), collected, and mixed with 1 mL of developer. Finally, the fumonisin concentration in the grain was quantified using the FumoniTestTM and the Fluorometer VICAM, following the manufacturer’s protocols (Jorge et al., 2022).

### 2.2 Pedigree and genotypic data

Pedigree and genomic information were available for a total of 246 hybrids originated from 236 unique inbred lines (Table 1) belonging to two heterotic groups: 142 inbred lines from the Flint group, and 94 inbred lines from the Dent group (see Supplementary Figure S1 for more details about the heterotic groups). The inbred lines were divided into two groups of parents: P1, 30 inbred lines used as male; and P2, 211 inbred lines used as female. A few inbred lines (only 5) were used as both male or female; thus, these genotypes are found in both P1 and P2 groups.

**TABLE 1:** Dataset summary of the availability of pedigree and genomic information for Hybrids, and groups of parents (P1, and P2).

Information	Hybrids	P1	P2
Pedigree	127	113	21
Pedigree and genomic	246	30	211
Total	373	143	232

For genotyping, the young leaves of 236 inbred lines were used for extracting genomic DNA via the CTAB method (Hexadecyltrimethylammonium bromide; Saghai-Maroof et al., 1984). The DNA quantification was done using a fluorometer, following the manufacturer’s instructions. The samples were shipped to the Genomic Diversity Facility of Cornell University (Ithaca, NY, United States) for genotyping-by-sequencing (GBS; Elshire et al., 2011). Using the Burrows-Wheeler alignment (BWA) tool (Li and Durbin, 2009), the sequences were aligned with the B73 reference genome (AGPv3). A total of 474,367 SNPs markers were available for analysis.

After applying quality control using TASSEL v.5.2.10 software (Bradbury et al., 2007), discarding those molecular markers with a Minor Allele Frequency (MAF) smaller than 10%, and a heterozygote’s proportion per locus above 10%, 73,083 polymorphic SNPs remained in the analyses. In addition, the SNPs with missing information were imputed using Beagle software (Browning and Browning, 2016). The SNP markers of the inbred lines were encoded as 0, 1, and 2, with 0 representing the allele with the minor frequency, one for the heterozygous, and two for the allele with the major frequency. The genotypes of the synthetic hybrids were built by combining the markers from the respective parental inbred lines using the expected value, which represents the mean allele dosage across parents for each marker.

In addition, there were also available phenotypic and pedigree information for an extra set of 127 hybrids totaling 373 when combined with the initial set of 246 hybrids that have both pedigree and genomic data (Table 1). This set of 127 hybrids was originated from 131 inbred lines belonging to three heterotic groups: 74 inbred lines from the Flint group, 49 inbred lines from the Dent group, and four inbred lines from the C group. The C group is intermediate to Flint and Dent, representing lines of several origins. It is well known there is a good combining ability between genotypes from the C group crossed with Dent or Flint testers (groups) (Silva et al., 2020). There was also information available regarding the pedigree of the ancestors of the inbred lines. Hence it was possible compute the pedigree-based relationship matrix for the hybrids ( $A_{H}$ ) and the corresponding matrices for the group of parents P1 ( $A_{P1}$ ), and P2 ( $A_{P2}$ ). It is worth noting that over 3 years, in the same experiment, there were hybrids whose parents were genotyped (246 hybrids) and hybrids whose parents were not genotyped (127 hybrids), due to limited resources of the breeding program.

### 2.3 Relationship matrices

The pedigree data was used to build the additive relationship matrix for P1 ( $A_{P1}$ ), P2 ( $A_{P2}$ ), and the hybrids ( $A_{H}$ ), according to Henderson (1976), which utilizes genealogical information to estimate genetic relatedness. This method is widely used in animal and plant breeding to quantify the genetic similarity among individuals based on their shared ancestry. The R package AGHmatrix (Amadeu et al., 2016) was used to build the additive relationship matrices for P1 ( $A_{P1}$ ), P2 ( $A_{P2}$ ), and the hybrids ( $A_{H}$ ). The genomic data was used to compute the realized genomic relationship matrix (or **G** matrix) for P1 ( $G_{P1}$ ), P2 ( $G_{P2}$ ), and the hybrids ( $G_{H}$ ) following VanRaden et al. (2008). VanRaden’s method estimates genetic relatedness based on actual genetic marker data, providing a more precise measure of genetic similarity by considering the genetic variation at specific loci across the genome. In addition, *a*) to having a better characterization of the matrix of genomic relationships, and *b*) to increasing the training set size by including non-genotyped inbred lines, *c*) the single-step relationship matrices were built for P1, P2, and the hybrid components to include information of the ancestry of the parents as in Khanna et al. (2022).

In general, the matrix that combines genomic and pedigree data is commonly named the **H** matrix (Velazco et al., 2019); however, since this research involves the prediction of hybrids, the use of **H** was reserved for denoting the genomic relationship matrix computed with the synthetic matrix of marker SNPs (mean of the allele dosage of the parents at each marker position). For this reason, the resulting matrix from the single-step procedure is named **B**-matrix. For example, the E + H model represents a linear predictor that includes the main effect of the environments and the main effect of the synthetic hybrid markers obtained as the mean across the marker information of the two parents involved in a specific crossing. This change was intended for an easier understanding for the reader. On the other hand, generically, the **B** (single step) matrix was built following Aguilar et al. (2010):

$$B=w\times A+\left(1-w\right)\times G$$ (1)

where **A** is the pedigree relationship matrix, **G** is the genomic relationship matrix, and w is the weighting factor (i.e., the fraction of total additive variance not addressed by the markers; Velazco et al., 2019). Here, the **B**-matrix in Equation 1 can be computed considering different sources of information. For example, combining the **H** and the **A** matrices, or the $G_{P1}$ and the $A_{P1}$ matrices, the $G_{P2}$ and the $A_{P2}$ matrices, and their corresponding interactions with environments, etc. Further details about all the different combinations and model terms considered in this study are below.

### 2.4 Prediction models

A two-stage approach was considered to implement the prediction models. In the first stage, within-environment adjusted means (best linear unbiased estimation–BLUE) were obtained with the following model:

$$y_{ikl}=\mu +h_{i}+r_{k}+b_{l\left(k\right)}+{ɛ}_{ikl}$$ (2)

where $y_{ikl}$ is the phenotypic value of the i ^th^ hybrid within the l ^th^ block of the k ^th^ replicate; μ is the constant, $h_{i}$ is the fixed effect of *i*th hybrid ( i = 1, 2, ., 373), $r_{k}$ is the fixed effect of replication k ( k = 1, 2), $b_{l\left(k\right)}$ is the random effect of the l ^th^ block within the k ^th^ replicate ( l = 1, 2, … ,16), with $b_{l\left(k\right)}\;∼\;N\left(0,\sigma_{b}^{2}\right)$ , where $\sigma_{b}^{2}$ is the block variance, and ${ɛ}_{ikl}$ is the residual random effect with ${ɛ}_{ikl}\;∼\;N\left(0,\sigma_{ɛ}^{2}\right)$ , and $\sigma_{ɛ}^{2}$ as the residual variance. A descriptive statistical analysis of the dataset was performed to assess the variance components and FUMO heritability for each environment and the results are presented in Supplementary Table S1. The analysis of the variance components employed a similar model to Equation 2 with the genetic effect of the hybrids considered as random such that $h_{i}\;∼\;N\left(0,\sigma_{h}^{2}\right)$ , where $\sigma_{h}^{2}$ represents the corresponding variance component. The first stage analysis was carried out using the statistical package ASReml-R v.4 (Butler et al., 2018) implemented in the statistical software R v.4.1.3 (R Core Team, 2022).

**FIGURE 2:**
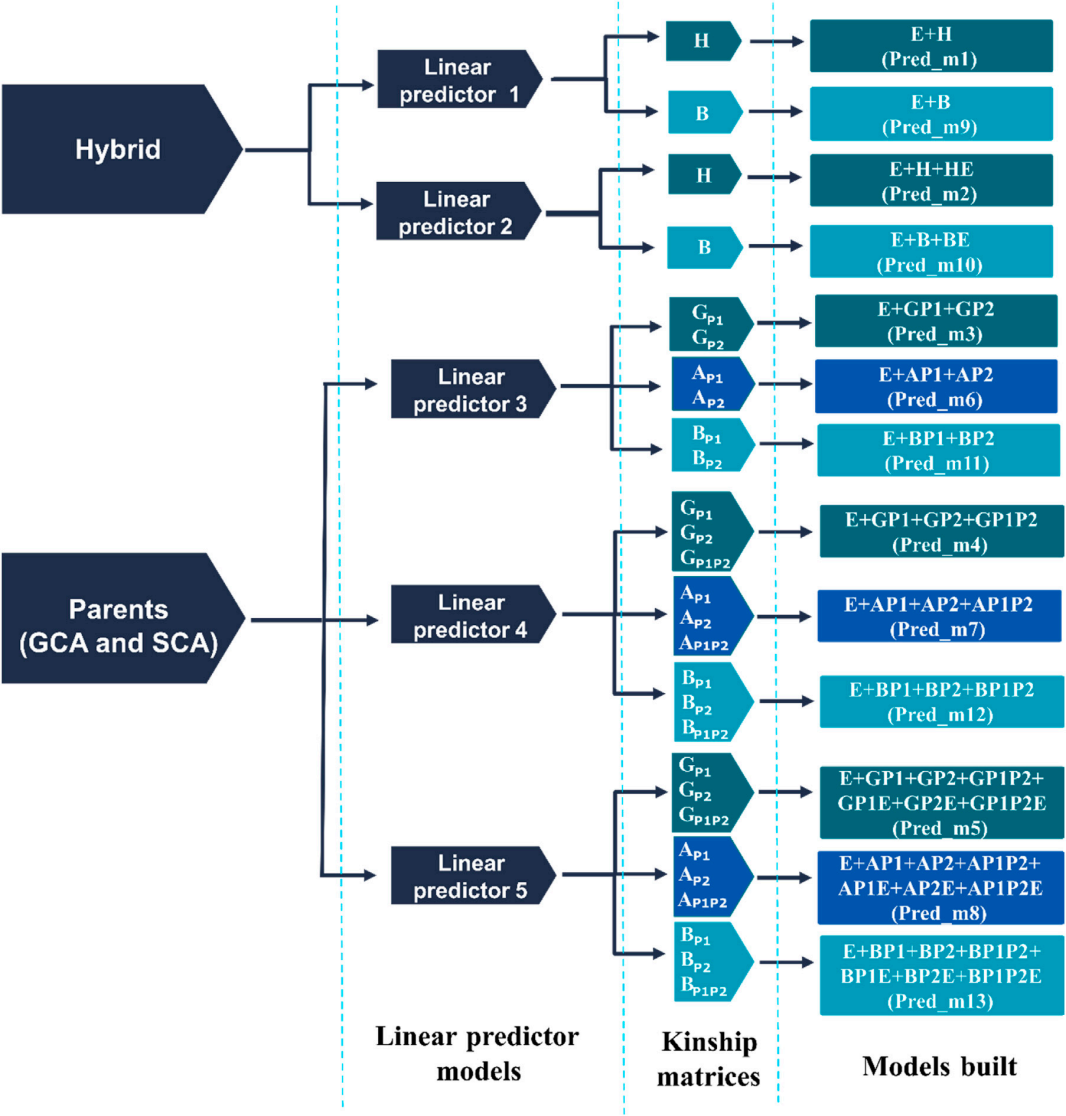
Prediction models derived from five linear predictors and four kinship matrices (**H**, **G**, **A**, and **B)**. Where H is the genomic relationship matrix of the hybrids based on synthetic marker SNPs, $G_{P1}$ is the genomic relationship matrix of the P1 group; $G_{P2}$ is the genomic relationship matrix of the P2 group; $G_{P1P2}=G_{P1}{}^{\circ}\;G_{P2}$ , $A_{P1}$ is the pedigree relationship matrix of the P1 group, $A_{P2}$ is the pedigree relationship matrix of the P2 group, $A_{P1P2}=A_{P1}{}^{\circ}\;A_{P2}$ , B is the single-step relationship matrix of the hybrid; $B_{P1}$ is the single-step relationship matrix of the P1 group, $B_{P2}$ is the single-step relationship matrix of the P2 group; $B_{P1P2}\text{ is the }B_{P1}{}^{\circ}\;B_{P2}$ , and; ° is the Hadamard or Shur product. The first two linear predictors (1 and 2) were implemented to model the hybrid performance involving the **H** matrix alone or in combination with pedigree via the single-step **B**-matrix. The other three linear predictors (3–5) considered the parents’ genomic and/or pedigree information via the general and specific combining ability (GCA and SCA) terms.

In the second stage, five different linear predictors were considered to model the trait response, and three different ways to account for relationships between hybrids using genomic (**H, G**_P1_, **G**_P2_), pedigree (**A**_P1_, **A**_P2_, **A**_P1P2_), and the single-step (**B**, **B**_P1_, **B**_P2_, **B**_P1P2_) information. Thirteen different prediction models resulted from combining the five linear predictors and the three different ways to introduce the relationships between hybrids (Figure 2; Supplementary Table S2). In principle, the first two linear predictors were implemented to model the hybrid performance directly, considering *i*) the main effects of the synthetic markers and *ii*) their corresponding interactions with environments. This model was also implemented to model the trait response using the pedigree matrix **A** of the hybrids and their corresponding interaction with environments. Hence, four different models can be constructed by combining the two linear predictors and the two ways to directly model the hybrids (M1, M2, M9, and M10 in Figure 2; Supplementary Table S2).

The other three linear predictors (3, 4, and 5) were considered to indirectly model the hybrids via the general and specific combining ability (GCA and SCA) terms. The GCA (P1+P2) term attempts to model the average effect of the parents involved in the crosses, while the SCA (P1 × P2) model term corresponds to the interaction of these effects. Similarly to the previous models, different sources of information were used to model the effects of the parents involved in the crosses: *1*) molecular marker information ( $G_{P1}$ , $G_{P2}$ , and $G_{P1P2}$ ), *2*) pedigree data ( $A_{P1}$ , $A_{P2}$ , and $A_{P1P2}$ ), and *3*) the combination of these sources of information using the single-step method: $B_{P1}={w\times A}_{P1}+\left(1-w\right)\times G_{P1}$ , $B_{P2}={w\times A}_{P2}+\left(1-w\right)\times G_{P2}$ , and $B_{P1P2}={w\times A}_{P1P2}+\left(1-w\right)\times G_{P1P2}$ . Further details are provided below to describe the election of the optimum value of the *w* hyper-parameter. In addition, the interactions of these terms with environments were also considered resulting in models M3, M4, M5, M6, M7, M9, M11, M12, and M13 (Figure 2; Supplementary Table S2).

Below, the linear predictors (1–5) and their corresponding components (Figure 2; Supplementary Table S2) are described.

#### 2.4.1 Linear predictor 1

Consider that ${\bar{y}}_{ij}$ represents the phenotypic response (FUMO) of the *i* ^th^ hybrid observed in the *j* ^th^ environment; depending on the different sources of information it can be modeled as follows:

$${\bar{y}}_{\mathrm{ij}}=\mu +E_{j}+k_{i}+{ɛ}_{\mathrm{ij}}$$ (3)

where μ is the general mean (constant effect across genotypes and environments), $E_{j}$ is the fixed effect of *j* ^th^ environment, $k_{i}$ models the random effect of the *i* ^th^ hybrid, where $k=\left\{k_{i}\right\}∼\text{MVN}\left(0,{K\sigma }_{k}^{2}\right)$ . The $k_{i}$ can be modeled considering different sources of information. For example, K could represent the covariance structure computed with the synthetic SNPs markers **H,** or the **B**-matrix of the hybrids combining **A** and **H** matrices as $w\times A+\left(1-w\right)\times H$ , with $\sigma_{k}^{2}$ being the additive variance, and ${ɛ}_{ij}\;∼\;N\left(0,\sigma_{ɛ}^{2}\right)$ , where $\sigma_{ɛ}^{2}$ is the residual variance. Thus, the possible models of this linear predictor described in Equation 3 are M1, and M9 (Figure 2).

#### 2.4.2 Linear predictor 2

This model is similar to linear predictor 1, but it also includes the interaction effect between hybrids and environments (G × E) via the reaction norm model (Jarquin et al., 2014). Considering the specific response of the *i* ^th^ hybrid in the *j* ^th^ environment through the model term ${kE}_{ij}$ , this linear predictor can be described as:

$${\bar{y}}_{ij}=\mu +E_{j}+k_{i}+{kE}_{ij}+{ɛ}_{ij}$$ (4)

where ${kE}_{ij}$ is the random effect of the hybrid-by-environment interaction such that $kE=\left\{{kE}_{ij}\right\}\;∼\text{ MVN}\left[0,\left(Z_{g}KZ_{g}^{\prime }\right){}^{\circ}\;\left(Z_{E}Z_{E}^{\prime }\right)\sigma_{kE}^{2}\right]$ , $Z_{g}$ and $Z_{E}$ are the incidence matrices that connect phenotypes with genotypes and environments, respectively, $\sigma_{kE}^{2}$ represents the variance component of the hybrid-by-environment interaction, and ° is the Hadamard or Shur product (the cell-by-cell product) between two matrices. Similar to the previous model in Equation 4, different sources of information (**H**, or **B**-matrix) can be used to model the G × E interaction term. In this case, the resulting models of this linear predictor are M2 and M10 (Figure 2).

#### 2.4.3 Linear predictor 3

This linear predictor was built by modeling the general combining ability (GCA) of the male and female parents involved in the crosses of the hybrids. Consider the *i*th hybrid originated from crossing parent 1 (P1) and parent 2 (P2), with $k_{P1i}$ and $k_{P2i}$ representing their corresponding effects. The following linear predictor was implemented to modeling the performance of the *i* ^th^ hybrid in the *j* ^th^ environment via the GCA of the inbred lines:

$${\bar{y}}_{ij}=\mu +E_{j}+k_{P1i}+k_{P2i}+{ɛ}_{ij}$$ (5)

where $k_{P1}=\left\{k_{P1i}\right\}∼\text{MVN}\left(0,{K_{P1}\sigma }_{P1}^{2}\right)$ , with $K_{P1}$ representing the kinship matrix of the P1 parent, $\sigma_{P1}^{2}$ being the associated variance component; $k_{P2\;}=\left\{k_{P2i}\right\}∼\text{ MVN}\left(0,{K_{P2}\sigma }_{P2}^{2}\right)$ , $k_{P2}$ representing the kinship matrix of the P2 parent, and $\sigma_{P2}^{2}$ being the associated variance component. Similar to the previous models in Equation 5, different sources of information can be used to model the relationship matrices for parents P1 ( $K_{P1}$ ) and P2 ( $K_{P2}$ ). For example, these can be modeled using the genomic information of the parental inbreds ( $G_{P1}$ , and $G_{P2}$ ), the pedigree information of the parental inbreds ( $A_{P1}$ , and $A_{P2}$ ; as in Khanna et al., 2022) or the combination of these components via the **B** matrices ( $B_{P1}$ , and $B_{P2}$ ). The resulting models of this linear predictor described in Equation 6 are M3, M6, and M11 (Figure 2).

#### 2.4.4 Linear predictor 4

This linear predictor is similar to the linear predictor 3; however, in addition to the GCA of the inbred lines it also includes the specific combining ability (SCA) of the parents involved in the corresponding crossing. The SCA was modeled as the interaction effect between the pair of parents according to Acosta-Pech et al. (2017). Combining the assumptions from the linear predictor 3 with the SCA term, the resulting linear predictor is:

$${\bar{y}}_{ij}=\mu +E_{j}+k_{P1i}+k_{P2i}+k_{P1P2i}+{ɛ}_{ij}$$ (6)

where $k_{P1P2}=\left\{k_{P1P2i}\right\}∼\text{MVN}\left(0,{K_{P1P2}\sigma }_{P1P2}^{2}\right)$ , with $K_{P1P2}=K_{P1}{}^{\circ}\;K_{P2}$ , and $K_{P1}$ and $K_{P2}$ being as previously described in the linear predictor 3, and $\sigma_{P1P2}^{2}$ is the associated variance component with this interaction term. This interaction term can be modeled using the genomic information of the parental inbreds, the pedigree information of the parental inbreds or the combination of these via the **B** matrices. The resulting models of this linear predictor are M4, M7, and M12 (Figure 2).

#### 2.4.5 Linear predictor 5

One of the disadvantages of the previous linear predictor is that it returns the same estimated predicted value for each genotype across environments. In addition, many of the hybrids share a common parent (either P1 or P2) but observed in other environments. In these cases, it is possible borrow information between hybrids sharing a common parent across environments. Acosta-Pech et al. (2017) and Jarquin et al. (2021) showed that inclusion of the GCA and SCA components significantly increase maize hybrid prediction. The resulting linear predictor is an extension of the fourth linear predictor that also includes the interaction between the components of the GCA ( $k_{P1},k_{P2}$ ) and SCA ( $k_{P1P2}$ ) terms with the environment via the reaction norm model. The resulting linear predictor is as follows:

$${\bar{y}}_{ij}=\mu +E_{j}+k_{P1i}+k_{P2i}+k_{P1P2i}+{kE}_{P1i}+{kE}_{P2i}+{kE}_{P1P2i}+{ɛ}_{ij}$$ (7)

where ${kE}_{P1}=\left\{\;{kE}_{P1i}\right\}\;∼\text{ MVN}\left[0,\left(Z_{P1}K_{P1}Z_{P1}^{\prime }\right){}^{\circ}\left(Z_{E}Z_{E}^{\prime }\right)\sigma_{P1e}^{2}\right]$ , ${kE}_{P2}=\left\{{kE}_{P2i}\right\}\;∼\text{ MVN}\left[0,\left(Z_{P2}K_{P2}Z_{P2}^{\prime }\right){}^{\circ}\left(Z_{E}Z_{E}^{\prime }\right)\sigma_{P2e}^{2}\right]$ , ${{kE}_{P1P2}=\{kE}_{P1P2i}\}\;∼\text{ MVN}\left[0,\left(Z_{P1}K_{P1}Z_{P1}^{\prime }\right){}^{\circ}\left(Z_{P2}K_{P2}Z_{P2}^{\prime }\right){}^{\circ}\left(Z_{E}Z_{E}^{\prime }\right)\sigma_{P1P2e}^{2}\right],$ $Z_{P1}$ , and $Z_{P2}$ are the incidence matrices that connect phenotypes with inbred parents P1 and P2; $\sigma_{P1e}^{2}$ , $\sigma_{P2e}^{2}$ and $\sigma_{P1P2e}^{2}$ are the corresponding variance components of the interaction between the GCA (P1 and P2) and SCA (P1 × P2) terms with the environments *E*. Since different sources of information can be used to model the covariance structures $K_{P1}$ , and $K_{P2}$ , the resulting models of the linear predictor described in Equation 7 are M5, M8, and M13 (Figure 2).

### 2.5 Cross-validation and **B**-matrix optimization

**FIGURE 3:**
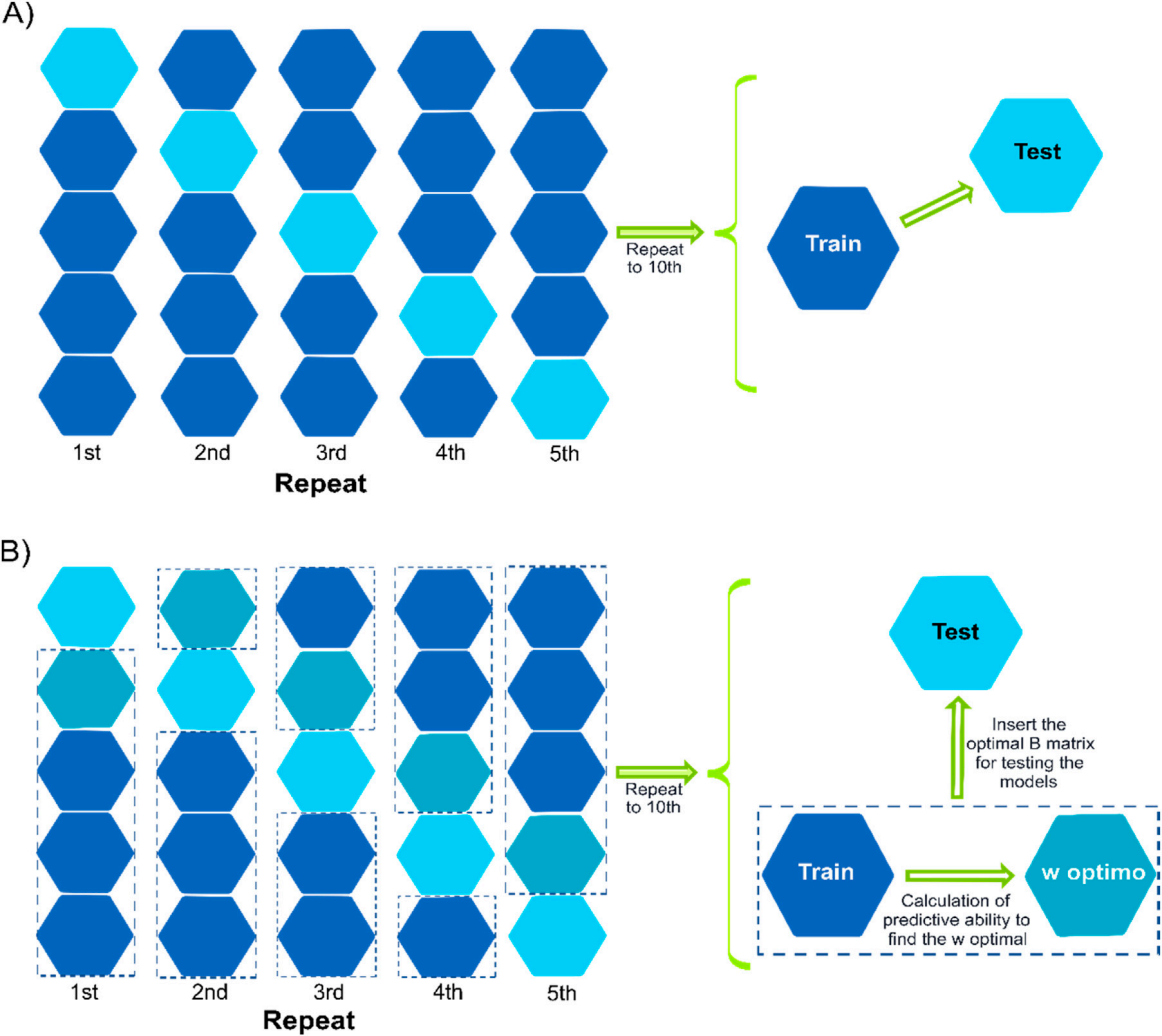
Representation of two cross-validation schemes with a five-fold assignation. Panel **(A)** describes the conventional cross-validation where four folds are used for model training to predict the fifth fold. In panel B, three folds are used for model training, and the fourth fold is used to optimize the election of the *w* value for those models involving the single-step **(B)** terms. Once the optimum values are found (across or within the environments of the fourth fold), the fifth fold is predicted. The columns represent the different replicates for assigning observations/genotypes to folds. The hexagons represent the folds, the blue hexagons correspond to the folds implemented as the training set, the blue-green hexagon corresponds to the fold used to conduct the optimization of the *w* hyper-parameter when the single-step model is implemented, and the cyan hexagon represents the testing fold (fifth fold).

For evaluating the model’s predictive ability, two cross-validation scenarios were implemented: *1*) CV1, which consists of predicting hybrids that have not been tested at any of the observed environments, and *2*) CV2, which consists of predicting the performance of hybrids that have been tested in some environments but not in others (incomplete field trials). Furthermore, two different ways of composing training sets represented in Figure 3 were considered. The traditional five-fold cross-validation scheme was implemented for the models that only use **H**, **G**, or the **A** matrices (models M1-M8; Figure 3A). This consists of a random five-fold (represented in Figure 3 by the hexagon), where four folds were used for model training, and the remaining fold was used as a testing set for evaluating the predictive ability. In this case, around 20% of the hybrids were predicted using 80% of the observed phenotypes. Ten replicates were considered to randomly assign phenotypes (CV2)/genotypes (CV1) to folds.

A slightly modified cross-validation approach was employed for the models incorporating the **B**-matrix (five models M9-M13), as depicted in Figure 3B. While maintaining the same five-fold partition, an optimization procedure was introduced to select the optimal *w* value to combining both covariance structures. In this setup, three folds were utilized for training the model, and the fourth fold was used to determine the optimum *w* value that yields the highest correlation between predicted and observed values. Regarding the fourth fold, a sequence of 21 values ranging from 0 to 1 (with increments of 0.05) for *w* was considered for constructing $B=w\times A+\left(1-w\right)\times G$ matrices. After fitting the models using three folds, the correlation between predicted and observed values on the fourth fold was computed. The *w* value that returned the highest correlation was chosen to construct the **B**-matrix for predicting the fifth fold (testing set). Subsequently, the last fold was employed to assess the predictive performance of the models (Figure 3B). This approach allocated approximately 60% of the phenotypic data for model training, 20% for *w* optimization, and another 20% for evaluating the model’s predictive ability given the optimized *w* value obtained at the fourth fold.

The correlation between predicted and observed values was computed on a trial basis (within environments); thus, the choice of the *w* value can be determined using two approaches: *1*) selecting the optimum value of *w* across environments and *2*) selecting the optimum value of *w* for each environment. For the first case, the weighted average correlation across the three environments was computed following Tiezzi et al. (2017) (see Equation 8 for more details), then the *w* value returning the highest average correlation was used for predicting the fifth fold. For the second case, the *w* value that returned the highest correlation between predicted and observed values for each environment was selected for predicting the remaining fifth fold. Therefore, up to three different *w* values could result from selecting the optimum *w* value for each environment. This procedure was repeated 10 times for each one of the cross-validation scenarios (CV1 and CV2). The statistical analyses were performed in R statistical package version 4.2.1 (R core team 2022), using the package BGLR, version 1.1.0 (Pérez and De Los Campos, 2014).

### 2.6 Training set composition

The single-step model has shown improvements in predictive ability in plant breeding implementations (Legarra et al., 2009; Cappa et al., 2019; Oliveira et al., 2020). These improvements are attributed to the fact that *i*) combining genomic and pedigree information potentially results in a better matrix describing relationships between pairs of individuals, and *ii*) the ability to increase the training set size for model calibration (Oliveira et al., 2020). This study considered two scenarios for composing training sets to assess the factors contributing to improving predictive ability when considering the single-step model (**B**). The first scenario, named genomic only dataset (GOds), consists of only individuals with genomic and pedigree information (246 hybrids). While the second scenario, named pedigree and genomic dataset (PGds), increases the training set size by including phenotypic information of individuals with pedigree data (373 hybrids). Some of these have both genomic and pedigree information. For the two training set composition scenarios, in principle, the same training set partitions described in Figure 3 were considered; however, for the second scenario, the training set was augmented with the phenotypic information of non-genotyped inbreds for whose pedigree information was available to connect with genotyped inbreds.

### 2.7 Within and across environments predictive ability

The predictive ability was assessed on a trial basis by computing Pearson’s correlation between predicted and observed values within the same environment. The average correlation across environments was calculated by accounting for uncertainty and the sample size of the environments (Tiezzi et al., 2017) as follows in Equation 8:

$$r_{\phi }=\frac{\sum_{j=1}^{J}\frac{r_{j}}{V\left(r_{j}\right)}}{\sum_{j=1}^{J}\frac{1}{V\left(r_{j}\right)}}$$ (8)

where $r_{j}$ is the Pearson’s correlation between the predicted and the observed values at the *j* ^th^ environment, $V\left(r_{j}\right)=\frac{1-{r_{j}}^{2}}{n_{j}-2}$ corresponds to the sampling variance, and $n_{j}$ is the corresponding number of observations. Furthermore, the across-environments mean squared prediction error (MSPE) was also computed as described in Equation 9.

$$MSPE=\frac{\sum_{j=1}^{J}\sum_{i=1}^{I}\left(y_{ij}-{\hat{y}}_{ij}\right)}{Total\;phenotypes}$$ (9)

where $y_{ij}$ is the phenotypic value of the *i* ^th^ hybrid at the *j* ^th^ environment, and ${\hat{y}}_{ij}$ is the corresponding predicted value using the above described 13 models (M1-M13).

### 2.8 Summary of the prediction strategies

Two cross-validation scenarios (CV1 and CV2) were considered to assess the predictive ability of the prediction models. A total of 13 prediction models (M1-M13) were derived from the five linear predictors and the three different sources of information to modeling the different model terms. For models M1-M8, the training set was composed of four folds, while the fifth fold corresponds to the testing set. On the other hand, the models M9-M13 involve single-step parameterization. Thus, these require an optimization process to select the value of the *w* hyper-parameter that returns the highest predictive ability using three folds for model calibration and the fourth fold to evaluate different values for *w* (21 values ranging from zero to one in steps of 0.05). In both groups of models (M1-M8, and M9-M13), the same partitions were considered for a direct comparison.

In addition, for all models using only genotypes with genomic data (GOds) for composing the training sets, an alternative was considered by augmenting the four folds with phenotypic information of inbreds with pedigree data (PGds) only. Thus, in this case, it is possible to evaluate whether the improvements in predictive ability are because *i*) a better relationship matrix is computed when combining genomic and pedigree data, or *ii*) direct benefits of increasing the training set by including phenotypic information of non-genotyped inbreds, or *iii*) interaction of both events occurring at the same time.

## 3 Results

### 3.1 Optimization of the **B**-matrix

**FIGURE 4:**
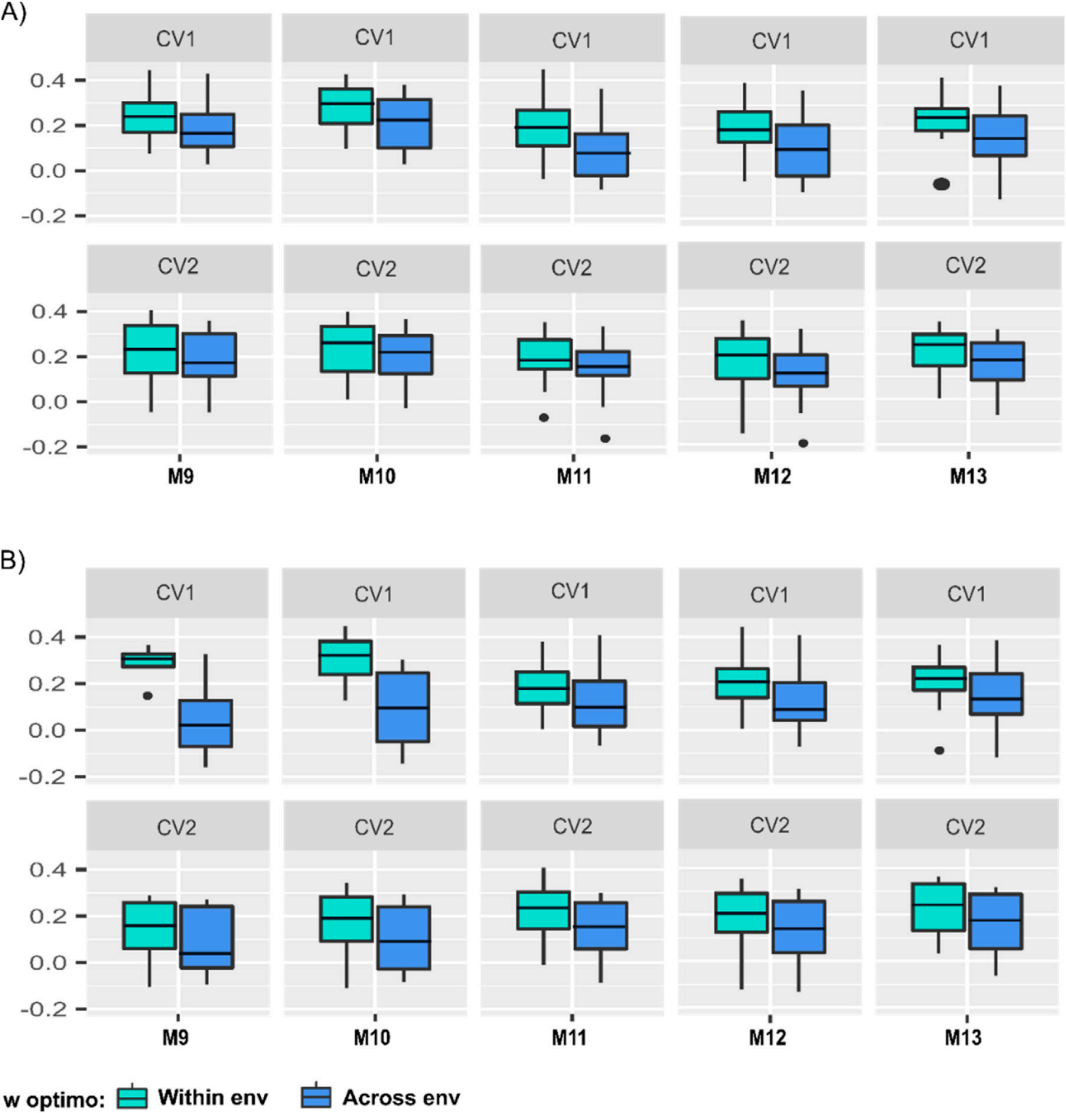
Boxplot of the predictive abilities of five linear predictor models in two prediction scenarios (CV1 and CV2), using two approaches to find the optimum *w* parameter of the **B**-matrix for models M9-M13. In blue color are indicated the results from selecting the *w* optimum value across environments, while in green, the results from selecting the within environments *w* value for the calibration sets: **(A)** the GOds (genomic-only dataset), and **(B)** the PGds (pedigree and genomic dataset). The numbers on the vertical y-axis are the models’ predictive abilities, and the horizontal x-axis represents models M9-M13.

Two different approaches were considered to find the optimum value of the *w* hyper-parameter used to blend/combine the **A** and the genomic (**H** or **G**) matrices according to the different linear predictors (1–5), the different ways for fitting the model terms (models M9-M13), and the different manners to compose calibration sets (i.e., GOds or PGds). The first case consists of optimizing *w* to return the highest weighted average correlation between predicted and observed values across environments, while the second approach focuses on finding the optimum *w* value for each environment such that up to three different values can be obtained. Figure 4 presents the weighted average (10 replicates) correlation across the three environments for both cross-validation schemes (CV1 and CV2), five prediction models M9-M13, two different ways to compose training sets (GOds and PGds), and both methods to find the optimum value of the *w* hyper-parameter (across and within environments).

As expected, when comparing these two approaches, for both prediction scenarios (CV1 and CV2) and both datasets (GOds and PGds), the predictive ability always improved when selecting *w* within environments for models M9-M13. Regarding the training set compositions, considering only information of genotyped individuals (GOds), under the CV1 cross-validation scheme, the average predictive ability ranged from 0.11 to 0.21, while between 0.09 and 0.18 for CV2 when performing the selection of the *w* value across environments (Figure 4A). On the other hand, when the *w* value was optimized within environments, the corresponding average predictive abilities increased, ranging from 0.18 to 0.26 (CV1) and 0.14 to 0.21 in (CV2; Figure 4A). The model that showed the highest predictive ability, for the two approaches (within and across environments), considering the optimization of the *w* hyper-parameter in both prediction scenarios (CV1 and CV2) and considering training sets of only genotyped individuals (GOds) was M10 (Figure 4A).

Augmenting the training set considering phenotypic information of individuals with pedigree data (PGds) when using the within environments *w* optimum value, the average predictive ability ranged from 0.20 to 0.32 and 0.12 to 0.21 for CV1 and CV2, respectively (Figure 4B). The model that showed the highest predictive ability for CV1 using *w* optimum across environments was M13. Nonetheless, when using the within environments *w* optimal, the highest predictive ability was shown with M10 (Figure 4B). These results indicate that the manner of computing the optimized **B**-matrix influences the election of the best prediction model. For CV2, the model that showed the best predictive ability, for both approaches (GOds, and PGds), within and across environments optimization, was M13. Due to the results presented, the *w* optimum value computed within environments was used on the models considering the **B** matrices (M9-M13) for comparing the predictive ability of the 13 models (M1-M13) for GOds and PGds training sets (see Figure 4).

### 3.2 Prediction models

**FIGURE 5:**
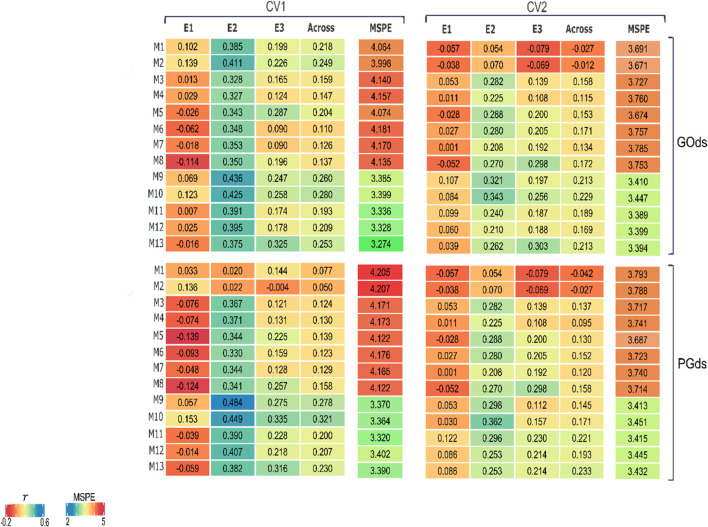
Heatmap of the predictive abilities of 13 models in two prediction scenarios (CV1 and CV2) to predict concentration of fumonisins (FUMO); r is the correlation between predicted and observed values, for each environment (E1 to E3) and across environments; MSPE is the mean squared prediction error across environments; GOds is the genomic-only dataset and; PGds is the pedigree and genomic dataset.

For an easy visualization, Figure 5 presents the heatmap of the within and across-environments predictive ability and the across-environments mean squared prediction error (MSPE) of the 13 models (M1-M13) for the two predicting scenarios (CV1 and CV2) and the two manners to compose training sets (GOds and PGds). As mentioned before, for models M9-M13, only the results derived from the within environments optimization are considered.

The predictive abilities varied considerably through environments for both prediction scenarios (CV1 and CV2) and both datasets used for model training (GOds, and PGds). Overall, there was an improvement in the across-environments weighted predictive ability and a reduction of the MSPE when the **B**-matrix was considered (higher correlations and reduced MSPE) in models M9-M13. Note that despite using phenotypic information from genotyped individuals (GOds) or combining it with also individuals with only pedigree data for increasing training set size (PGds), the models including the **B**-matrix improved the predictive ability compared with those that only used the genomic information (**H** or **G** matrices) in M1-M5 models. In general, the models presenting the best results, within and across environments, are those that considered the interaction between the genetic (**H**, **G**, **A**, or **B**) and the environmental component (E).

#### 3.2.1 CV1 scheme

In the scenario of predicting untested hybrids in evaluated environments (CV1), the across-environments predictive ability of the prediction models using GOds data to compose training sets ranged from 0.11 to 0.28 (Figure 5). In this case, the best prediction models for E1, E2, and E3 were M2 (0.139), M9 (0.436), and M13 (0.325), respectively. However, across environments, the M10 model (E + B + BE) showed the highest predictive ability (0.280) and a reduced MSPE (3.399) compared with most of the models. When the training sets were augmented with individuals with only pedigree data (PGds), the across-environments predictive ability ranged from 0.050 to 0.321 (Figure 5), with the models including the different **B** matrices showing the best results across environments. For PGds, the model with the best predictive ability across the environments was also M10 returning a correlation of 0.321 and a MSPE of 3.364. Note that a relative improvement in the predictive ability of around 15% (i.e., 0.280 vs. 0.321) was accomplished when the training set size was increased also including phenotyped individuals (PGds) with model M10.

#### 3.2.2 CV2 scheme

Contrary to what was initially expected (based on similar studies), in the scenario that predicts the performance of already observed hybrids in some environments but not in others, the predictive abilities were lower than those from the CV1 scheme (Figure 5), which is a more complex prediction problem. Thus, for this particular dataset, it is better to ignore the available information from the target hybrids observed in environments different from those of interest and assume these as totally unobserved hybrids across environments (CV1 scheme).

For the GOds manner of composing training sets, the across-environments predictive ability ranged from −0.027 to 0.229. For all environments, the models including the different **B** matrices showed the highest predictive ability; however, different prediction models showed the best results for each environment. The M10 showed the highest across-environments correlation (0.229) and a low MSPE (3.447). Regarding the PGds training sets, the predictive ability across the different environments ranged from −0.042 to 0.223. The corresponding models with the highest correlations for E1, E2, and E3 were M9 (0.107), M10 (0.343), and M8 (0.298). However, across environments, model M13 showed the highest correlation (0.233). In this prediction scenario, it was also observed that the across-environments results of the M13 model trained with GOds (0.213) dataset were improved when increasing the individuals in the training set using the PGds set (0.233). Note that, for this model, the correlation increased by 9.4% when using the PGds. In contrast, model M10 did not improve the predictive ability adding phenotypes of non-genotyped individuals (i.e., from Gods to PGOds), showing a reduction of ∼33.9% (0.229 vs. 0.171).

### 3.3 Impacts of combining genomic information and pedigree data

In the previous section, across environments, mixed results were shown with the increasing of the training set by adding those individuals with pedigree information only. For CV1, the best results were observed when the training set was augmented (0.321) via the PGds data set in comparison with the GOds set (0.280). Recalling that in both cases, for the most promising models (M10-M13), the genomic information and the pedigree data are combined via the **B** matrices (after an optimization process). However, the only difference is that for the GOds the training set is composed for only those individuals that have both genomic and pedigree data, while for the PGds the previous data set is augmented by adding the phenotypic information from individuals with only pedigree information.

**FIGURE 6:**
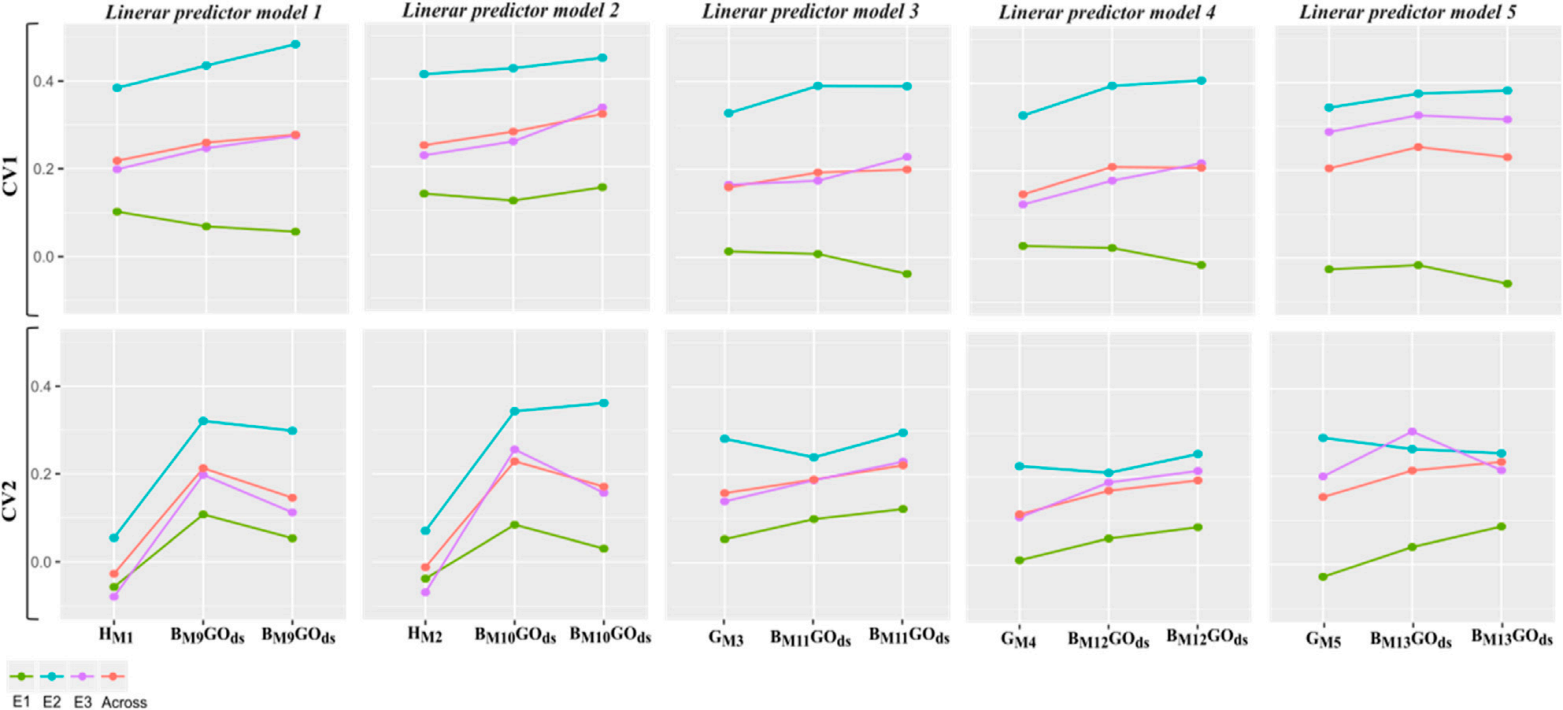
Within and across environments weighted mean average (10 replicates of a five-fold cross-validation) correlation between predicted and observed values (vertical y-axis) for five linear predictors and two different manners to model the different model terms (horizontal x-axis): *1*) considering only the genomic information of synthetic hybrids or from parental inbreds (M1-M5); *2*) combining genomic and pedigree data via the **B** matrices to model the relationship matrix between pairs of individuals (M9-M13). For the second case, two different approaches to compose calibration sets are considered: GOds which uses in training sets only genotypes with both pedigree and genomic information; and PGds which includes phenotypic information from genotypes with pedigree data only for increasing the calibration set sizes.

For CV2, an opposite trend was observed, with GOds returning better results (0.229) than PGds (0.171) when considering the most promising model (M10) from the previous cross-validation scenario. However, in this case, for PGds M13 returned the highest correlation (0.233). In any case, for M11-M13 models, slightly improvements were observed when using PGds vs. GOds. These differences might not be significant; thus, no clear advantages were observed with the increasing training set size as for the CV1 case. Figure 6 depics a direct comparison of the effects in predictive ability *i*) from combining genomic and pedigree information (GOds), *ii*) with also an increased training set size (PGds) with respect to the models based on only a single data type (**H**, **G** or **A** used to describe similarities between pairs of individuals).

More specifically, in Figure 6, the results of the within and across environments predictive ability of the five linear predictors are depicted for single **H** (M1-M2), **G** (M3-M5) and combined with **A** through **B** (M9-M13) covariance structures considering phenotypic information of only individuals with genomic and pedigree data (GOds), and adding information from individuals with only pedigree data (PGds).

Under the CV1 scheme, for the linear predictor 1, on average moderate improvements are observed by combining the matrices **H** and **A** (especially for E2) compared with only using the **H** matrix to model relationships between pairs of individuals (M9 vs. M1). Also, in average, slight improvements were observed when the training set size was increased with individuals with only pedigree data (PGds) compared to the case where only the phenotypic information of individuals with both genomic and pedigree data (GOds) was used for model calibration.

A similar pattern was observed for the second linear predictor, where the interaction with environmental factors was included using the same covariance structures (**H** vs. **B**) contrasting models M2 and M10. In addition, the linear predictors 3-5 showed similar patterns. In all the cases, very low correlation values were observed for E1, suggesting this environment’s high influence contributing to the G × E interaction.

Regarding the CV2, more pronounced improvements in predictive ability were found by combining **H** and **A** matrices for linear predictors 1 and 2. However, these decreased when the information from non-genotyped was also included in the calibration sets, except for E2, where it practically remained the same. For the linear predictors 3 and 4, some improvements were observed with the PGds compared to GOds, while with the most complex linear predictor (5), mixed results were found depending on the environment.

## 4 Discussion

### 4.1 **B**-matrix optimization

This study introduces an approach for optimizing the hyper-parameter *w* using a novel method. To find the optimum *w* value, a cross-validation strategy using four of the original five folds was implemented (three folds for model fitting and one-fold to optimize *w* for 21 different values evenly spaced between 0 and 1 in steps of 0.05). Then the phenotypic information of the fifth fold was predicted considering the obtained *w* optimum value. This strategy offers a robust method for selecting the hyper-parameter *w*, returning satisfactory results regarding the accuracy of genomic predictions, which is particularly crucial for complex traits such as FUMO. This suggests that using the fourth fold for **B**-matrix optimization can be a good strategy to guide the election of the w hyper-parameter.

Available studies have shown that the election of the *w* optimum value varies according to the different characteristics being evaluated (Liu et al., 2011; Ashraf et al., 2016; Velazco et al., 2019). Nonetheless, our study presents a significant contribution by demonstrating the importance of selecting the optimal w by environment in unbalanced datasets, an approach not yet utilized in previous research. Until now, the studies have focused on selecting the *w* optimal value across environments (i.e., a common optimum value for all the environments). However, individuals have different genetic responses when changing environmental conditions, with non-static variation (e.g., temperature, precipitation, humidity) being the main source of genotype-environment interaction for maize (Cullis et al., 2000; Kleinknecht et al., 2013; Dias et al., 2020; Krause et al., 2022).

Our results reveal that, particularly in situations with unbalanced datasets, the difference in gene expressions of individuals between environments can significantly influence the election of the w optimal weighting factor. This finding has important implications for multi-environment and multi-trait studies, suggesting that researchers should consider not only the selection of *w* by trait, as is customary, but also by environment. The change in the value of *w* with unbalanced data occurs due to the alteration in the relative contribution of genomic and pedigree information to the genetic structure, which may necessitate a reevaluation of the hyper-parameter *w* to ensure that the modeling of genetic relationships is accurate and appropriate for the specific data in question. Consequently, our study demonstrates that the best strategy for building the **B**-matrix should be optimizing the *w* hyper-parameter within in environments, which is particularly relevant for crop improvement in tropical environments where G × E interactions are pronounced, and datasets are often unbalanced.

### 4.2 Prediction models

Over the decades, breeders have sought strategies to reduce the FUMO content in grain (Duvick, 2001; Eller et al., 2008; Pádua et al., 2016; Santiago et al., 2020; Butoto et al., 2022). The USFDA Center for Food Safety and Applied Nutrition (USFDA, 2021) recommends a maximum of 2–4 mg kg^-1^ of contamination of FUMO in corn products. In Brazil, the contamination limit is 2 mg kg^-1^ of these toxins in grains (Agência Nacional de Vigilância, 2011). Exceeding these limits prevents the exportation and national marketing of maize batches, leading to substantial economic losses for producers. Therefore, in addition to high grain yield performance, developing cultivars resistant to FUMO is a primary objective. However, phenotyping this trait is laborious and expensive (Bush et al., 2004). Consequently, implementing genomic selection becomes an important alternative strategy for developing fumonisin-resistant genotypes. Comparing genomic to traditional phenotypic selection for FUMO Butoto et al. (2022) found that both methods performed similarly. Nevertheless, the authors highlight that genomic selection has the potential to be more efficient than phenotypic selection due to the employment of cheaper and faster genotyping methods.

To our knowledge, no studies in the literature have combined genomic and pedigree information to construct relationship covariance structures for predicting FUMO. Our results demonstrate that the use of the **B**-matrix improved predictive ability in all tested linear predictors for both prediction scenarios and datasets (GPds and POds) compared to conventional GS models. This improvement in predictive ability is crucial for complex traits like fumonisin resistance, where accurate predictions can significantly impact breeding program efficiency. As observed, the use of a single-step approach was superior to the use of only the **H** matrix, regardless of whether the training set size was increased with un-genotyped individuals. Since some markers may not be in linkage disequilibrium with QTLs, when combining the **A** matrix and **H** or **G** matrix in a relationship the pedigree information may have contributed to capturing associations between causative alleles due to common ancestral identity, improving predictions models (Velazco et al., 2019). This finding has important practical implications, especially in situations where genotyping costs are a constraint. The **B**-matrix approach allows increase the size of the training set by including un-genotyped individuals through pedigree information, potentially leading to more robust and accurate predictions.

The observed improvements in prediction accuracy using the **B**-matrix align with findings from previous studies by Imai et al. (2019) and Cappa et al. (2019). These authors found that in terms of prediction accuracy, the **B**-matrix matched or surpassed the use of only the **G** matrix or the **A** matrix only. Additionally, over the years, several other authors have reported enhanced prediction accuracy with the use of the **B**-matrix in plant and animal species (Ashraf et al., 2016; Gao et al., 2012; Koivula et al., 2012; Lourenco et al., 2015; Pérez-Rodríguez et al., 2017). Furthermore, the single-step method allows the utilization of larger phenotypic datasets compared to the GBLUP method, as demonstrated herein for maize. Moreover, all the tested models corresponding to the different linear predictors reached the lowest MSPEs when using the **B**-matrix in comparison to those using only one data type to model similarities between pairs of individuals (**H**, **G**, or **A**). These results are consistent with those obtained by Velazco et al. (2019). The selection of prediction models based on minimizing MSPE has been recommended because this statistic considers both the precision and bias of the models (Vitezica et al., 2011; González-Recio et al., 2014).

Mainly in animal breeding, the use of the **B**-matrix in genomic prediction has been widely discussed (Martini et al., 2018; Macedo et al., 2020; Mäntysaari et al., 2020; Masuda et al., 2021). However, this methodology has not yet become very popular in plant breeding (Oliveira et al., 2020). Our results demonstrate the advantage of using the single-step approach in the intermediate stage of a breeding program in two different contexts. The first context is when the program does not have all individuals genotyped (PGds), for example, due to new materials inserted in the pipeline. In this case, the **B**-matrix enables the construction of the relationship matrix incorporating all individuals improving the predictive capacity of the models. The simultaneous use of genotyped and un-genotyped individuals relies on projecting genomic relationships to un-genotyped individuals based on the conditional distribution of breeding values for un-genotyped and genotyped individuals (Legarra et al., 2009). The genomic relationships can improve the pedigree relationships, while the un-genotyped individuals provide more phenotypic information. As a strategy, de Oliveira et al. (2020) used the **B**-matrix in multi-trait multi-environment genomic prediction models due to the lack of genotypic information for some evaluated maize hybrids. Likewise, Cappa et al. (2019) noted that the utilization of the single-step method resulted in elevated prediction accuracies and reduced bias of the genetic component of unobserved (non-phenotyped) but genotyped individuals compared to the standard GBLUP by using additional phenotypic information from non-genotyped individuals.

In our study, including phenotypes from un-genotyped individuals increased the predictive ability in most but not all the tested scenarios. In agreement with our findings, in a study with a broiler population, Hidalgo et al. (2021) found that the two most recent years of pedigree, phenotypic, and genomic data were sufficient to maintain prediction accuracies in selection candidates (i.e., the last generation of individuals), adding phenotypes of un-genotyped individuals from previous years did not increase the accuracy. Lourenco et al. (2014) stated that distant ancestors have minor contributions, explaining the null or marginal increase in predictive ability, and sometimes their inclusion can deteriorate predictive ability.

The second context consists of companies with genomic and pedigree information for all individuals (GOds). Our results showed that by combining both sources of information for modeling covariance structures, the predictive ability of the models increases, helping to prevent (discard) of advancing to the next stages of the program the most susceptible hybrids to FUMO. The increased predictive ability can be explained because the markers do not capture all the genetic variance; blending the genomic relationship matrix with a portion of the pedigree relationship matrix (with an optimal value w ) implicitly fits a residual polygenic effect in the statistical model via the modified genomic relationship matrix. Although the genomic relationship matrix accounts for most of the genetic variation, its combination with the pedigree relationship matrix increases the captured genetic variance. Furthermore, the combined use of pedigree and genomic information in the **B**-matrix can help mitigate the limitations of using the **G** matrix alone, such as incomplete linkage disequilibrium between markers and causal variants, especially in cases where the trait is influenced by rare or less common genetic variants, or imperfect genomic data. By leveraging the complementary information from both sources, the **B**-matrix provides a more comprehensive and accurate representation of the genetic relatedness among individuals, ultimately enhancing the predictive ability of the models. Other researchers have also observed that combining genomic and pedigree information optimizes genomic prediction for complex traits (Crossa et al., 2013; Basnet et al., 2019; Velazco et al., 2019).

Our study employed two cross-validation schemes, CV1 and CV2, to evaluate the predictive ability of various models. The CV1 scheme, which predicts untested hybrids in evaluated environments, generally showed higher predictive abilities compared to the CV2 scheme. This finding was contrary to our initial expectations based on similar studies in the literature (Jarquin et al., 2021; Khanna et al., 2022; Persa et al., 2021). One of the factors that could contribute to this unexpected outcome is the unbalanced nature of our dataset that could potentially impact the effectiveness of the CV2 approach, which relies on information from other environments. These findings underscore the need for further research to understand the factors influencing the relative performance of CV1 and CV2 schemes in different contexts, particularly for traits showing strong environmental influences. Finally, the breeding programs are also interested in developing superior cultivars that respond favorably to diverse environmental conditions (Jarquin et al., 2021). Our results were consistent with those obtained by several researchers that observed better predictive ability in models that consider genetic and environmental interaction effects (Jarquin et al., 2014; 2021; Lado et al., 2016; Basnet et al., 2019; Khanna et al., 2022), further emphasizing the importance of accounting for G × E interactions for reaching improved prediction accuracies of complex traits in tropical maize breeding programs.

## 5 Conclusion

The findings of this study highlight the importance of combining genomic and pedigree data. Particularly, optimizing the election of the *w* hyper-parameter to construct the **B**-matrix when dealing with diverse environments and unbalanced datasets. The most convenient optimization resulted when it was implemented within environments compared to across environments. This strategy improves the predictive ability of the models used for making predictions. The **B**-matrix was shown to enhance the predictive ability of the tested linear models for different prediction scenarios and datasets, compared to the **G** and, **A** matrices. Hence, the single-step approach helps improve the selection accuracy of FUMO trait.

## Data Availability

The original contributions presented in the study are included in the article/Supplementary Material, further inquiries can be directed to the corresponding author.
