# Supplementary material for: Optimizing the single-step model for predicting fumonisins resistance in maize hybrids accounting for the genotype-by-environment interaction
Source: Front Genet. 2025 Jul 2;16:1475452. doi: 10.3389/fgene.2025.1475452 (PMC12263360; doi:10.3389/fgene.2025.1475452)
Supplement: Supplementary file 1 [file Supplementaryfile1.doc]

Supplementary Material

**
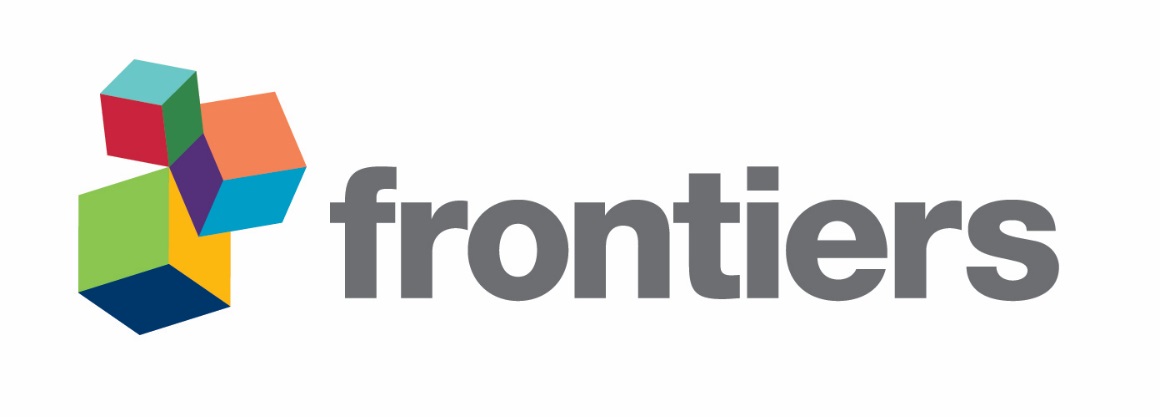
**

**Supplementary Table S1**. Estimates of variance components and heritability of FUMO over three environments.

| Parameters | **E1** | **E2** | **E3** |
| --- | --- | --- | --- |
| $\sigma_{h}^{2}$ | 0.80 | 2.41 | 2.11 |
| $\sigma_{b}^{2}$ | 0.00 | 0.10 | 0.15 |
| $\sigma_{ɛ}^{2}$ | 4.65 | 1.27 | 2.27 |
| $h_{h}^{2}$ | 0.15 | 0.64 | 0.47 |

$\sigma_{h}^{2}$: genotypic variance; $\sigma_{b}^{2}$: block variance; $\sigma_{ɛ}^{2}$: residual variance $h_{h}^{2}$: individual heritability; **E1:** Environment 1; **E2:** Environment 2; **E3:** Environment 3.

| **Model** | **Linear predictor type** | | | | |  | **Kinship matrix (K)** | | | | | | | | | | |
| --- | --- | --- | --- | --- | --- | --- | --- | --- | --- | --- | --- | --- | --- | --- | --- | --- | --- |
|  |  |  |  |  |  |  |  |  |  |  |  |  |  |  |  |  |  |
|  | 1 | 2 | 3 | 4 | 5 |  | $\mathbf{H}$ | $\mathbf{G}_{P1}$ | $\mathbf{G}_{P2}$ | $\mathbf{G}_{P1P2}$ | $\mathbf{A}_{\mathbf{P1}}$ | $\mathbf{A}_{P2}$ | $\mathbf{A}_{P1P2}$ | $\mathbf{B}$ | $\mathbf{B}_{P1}$ | $\mathbf{B}_{P2}$ | $\mathbf{B}_{P1P2}$ |
| M1: E+H | ● |  |  |  |  |  | ● |  |  |  |  |  |  |  |  |  |  |
| M2: E+H+HE |  | ● |  |  |  |  | ● |  |  |  |  |  |  |  |  |  |  |
| M3: E+G_P1_+G_P2_ |  |  | ● |  |  |  |  | ● | ● |  |  |  |  |  |  |  |  |
| M4: E+G_P1_+G_P2_+G_P1P2_ |  |  |  | ● |  |  |  | ● | ● | ● |  |  |  |  |  |  |  |
| M5: E+G_P1_+G_P2_+G_P1P2_+G_P1_E+G_P2_E+G_P1P2_E |  |  |  |  | ● |  |  | ● | ● | ● |  |  |  |  |  |  |  |
| M6: E+A_P1_+A_P2_ |  |  | ● |  |  |  |  |  |  |  | ● | ● |  |  |  |  |  |
| M7: E+A_P1_+A_P2_+A_P1P2_ |  |  |  | ● |  |  |  |  |  |  | ● | ● | ● |  |  |  |  |
| M8: E+A_P1_+A_P2_+A_P1P2_+A_P1_E+A_P2_E+A_P1P2_E |  |  |  |  | ● |  |  |  |  |  | ● | ● | ● |  |  |  |  |
| M9: E+B | ● |  |  |  |  |  |  |  |  |  |  |  |  | ● |  |  |  |
| M10: E+B+BE |  | ● |  |  |  |  |  |  |  |  |  |  |  | ● |  |  |  |
| M11: E+B_P1_+B_P2_ |  |  | ● |  |  |  |  |  |  |  |  |  |  |  | ● | ● |  |
| M12: E+B_P1_+B_P2_+B_P1P2_ |  |  |  | ● |  |  |  |  |  |  |  |  |  |  | ● | ● | ● |
| M13: E+B_P1_+B_P2_+B_P1P2_+B_P1_E+B_P2_E+B_P1P2_E |  |  |  |  | ● |  |  |  |  |  |  |  |  |  | ● | ● | ● |

**Supplementary Table S2**. Prediction models building blocks corresponding to five different linear predictor forms and different kinship matrices (**H**, **G**, **A**, and **B**).

M: prediction model; $H$: genomic relationship matrix of the hybrid; $G_{P1}$: genomic relationship matrix of the P1 group; $G_{P2}$: genomic relationship matrix of the P2 group; $G_{P1P2}: G_{P1}^{\circ} G_{P2}$; $A_{P1}$: pedigree relationship matrix of the P1 group; $A_{P2}$: pedigree relationship matrix of the P2 group; $A_{P1P2}: A_{P1}^{\circ} A_{P2}$; $B$: single-step relationship matrix of the hybrid; $B_{P1}$: single-step relationship matrix of the P1 group; $B_{P2}$: single-step relationship matrix of the P2 group; $B_{P1P2}: B_{P1}^{\circ} B_{P2}$; and $^{\circ}$ represents the Hadamard or Shur product.

**
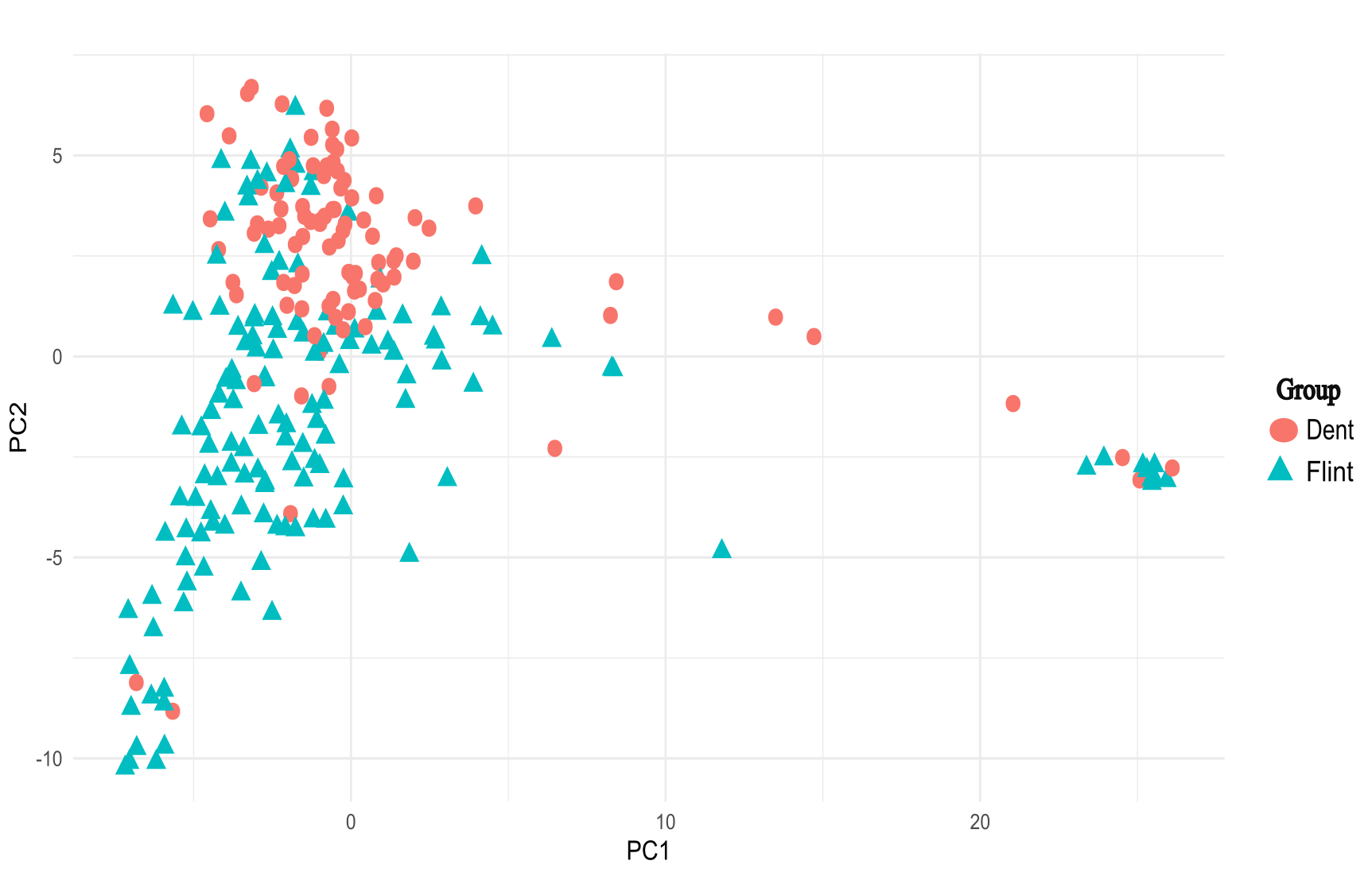
Supplementary Figure S1.** Principal component analysis for 236 genotyped inbred lines belonging to two heterotic groups: 142 inbred lines from the Flint group, and 94 inbred lines from the Dent group.
